# Factors predicting training transfer in health professionals participating in quality improvement educational interventions

**DOI:** 10.1186/s12909-017-0866-7

**Published:** 2017-01-31

**Authors:** Ahmed Eid, Doris Quinn

**Affiliations:** 1 Department of General Oncology, The University of Texas MD Anderson Cancer Center, 1515 Holcombe Blvd., Unit 0462, Houston, TX 77030 USA; 2 Consultant in Process Improvement, 7820 Friends Creek Rd, Emmitsburg, MD 21727 USA

**Keywords:** Change-driven culture, Health professionals, Quality improvement, Success case method, Training, Training transfer, Work environment

## Abstract

**Background:**

Predictors of quality improvement (QI) training transfer are needed. This study aimed to identify these predictors among health professionals who participated in a QI training program held at a large hospital in the United States between 2005 and 2014. It also aimed to determine how these predictive factors facilitated or impeded QI training transfer.

**Methods:**

Following the Success Case Method, we used a screening survey to identify trainees with high and low levels of training transfer. We then conducted semistructured interviews with a sample of the survey respondents to document how training transfer was achieved and how lack of training transfer could be explained. The survey’s response rate was 43%, with a Cronbach alpha of 0.89. We then conducted a thematic analysis of the interview transcripts of 16 physicians.

**Results:**

The analysis revealed 3 categories of factors influencing the transfer of QI training: *trainee characteristics*, *training course*, and *work environment*. Relevant trainee characteristics included *attitude toward change*, *motivation*, *mental processing skills*, *interpersonal skills*, and the personality characteristics *curiosity*, *humility*, *conscientiousness*, *resilience*, *wisdom*, and *positivity*. The training project, team-based learning, and lectures were identified as relevant aspects of the training course. Work culture, work relationships, and resources were subthemes of the *work environment* category.

**Conclusions:**

We identified several QI training transfer predictors in our cohort of physicians. We hypothesize that some of these predictors may be more relevant to QI training transfer. Our results will help organizational leaders select trainees who are most likely to transfer QI training and to ensure that their work environments are conducive to QI training transfer.

**Electronic supplementary material:**

The online version of this article (doi:10.1186/s12909-017-0866-7) contains supplementary material, which is available to authorized users.

## Background

Health care organizations and health professionals spend substantial amounts of time, effort, and funding on quality improvement (QI) training, yet there is little published evidence about the effectiveness of QI training for clinicians [1, 2]. Several studies and reviews have suggested that the impact of QI training may be mixed [3–7]. It is, therefore, important to understand the circumstances under which QI training is likely to be effective and, in particular, the optimal selection criteria for and characteristics of participants who are likely to successfully manage change in their departments and practices. Baldwin and Ford [8] defined training transfer in terms of 2 factors: (1) maintenance of newly acquired knowledge and skills and (2) application of newly acquired knowledge and skills to new areas. According to the authors, training transfer occurs when trainees finish the training session and apply what was learned to their work.

Studies have distinguished 3 groups of factors affecting training transfer at work: (1) factors related to the trainee, (2) factors related to the training program, and (3) organizational factors involving both the training program and the trainee [8–13]. The effects of trainee characteristics on training transfer have been investigated in many research studies and reviews [8, 9, 14–16]. In a recent review of the training literature, Salas et al. [15] included “person analysis” as one of the best practices for maximizing training effectiveness.

To our knowledge, however, no studies have identified the predictors of QI training transfer among health professionals. The goal of this study was to fill this gap in the literature by identifying the factors predicting QI training transfer among health professionals who participated in a QI training program held between 2005 and 2014 at a large health care facility in the south-central United States. This article describes the views of QI training participants and the impact participation in QI training had on the transfer of their training to their practices. The factors and personal attributes predicting QI training transfer were analyzed and described with the aim of helping organizational leaders select trainees who are most likely to transfer QI training and to ensure that their organizations’ work environments are conducive to QI training transfer.

## Methods

### QI training program

The QI training program studied here is funded by a state university system with which the host institution is affiliated. It is offered twice a year and consists of 8 full-day instruction sessions given over a period of 6 months. It requires participants to work in teams of at least 2 trainees and others from their practice area to complete a QI project. It is highly encouraged that each team include at least 1 faculty physician. Besides the physicians, the other participants on the teams include other health care professionals and administrative personnel who typically work together in their everyday environments. The course is voluntary, though division heads and department chairs can invite participants or request that individuals participate. At the conclusion of the program, the trainees are expected to have learned to (1) improve health care systems and processes; (2) identify, measure, and reduce variation in processes; (3) understand the need for multiprofessional and interdisciplinary teamwork in improving patient care; (4) improve health care outcomes; and (5) increase organizational learning by sharing knowledge of best QI practices. The training core components have not been modified over the years the course has been offered.

### Research design and sample selection

This study used the Success Case Method, which involves 2 steps: (1) identifying training successes and lack of successes, usually by a screening survey of trainees; and (2) understanding how the trainees’ successes were achieved or not achieved, usually by conducting interviews with a sample of the most and least successful trainees as identified using the survey [17]. The Success Case Method has previously been used to assess training in the health professions by Olson et al. [18].

An online screening survey was designed by both authors and reviewed for face and content validity by 2 health care quality experts, each with over 25 years of experience. The first 3 questions of the survey were designed based on the critical applications of the QI training program (Additional file 1: Appendix A). The answers to the survey questions were used as training transfer indicators in this research. In addition to asking the training participants about QI projects they developed since participating in the training, we also asked how the trainees applied the skills learned in the training in their practices and departments. The trainees’ descriptions of the QI projects were also verified during the interview process.

We used the training program database to identify all participants in the QI training courses given between 2005 and 2014. The survey was sent via email to the training participants who were still affiliated with the institution at the time of the study. Participants were asked to provide informed consent using the online survey tool, Qualtrics. The following sociodemographic variables with potential impact on training transfer were collected: age, gender, profession, academic role (if any), length of work experience, and training cohort. The responses were weighted as shown in Additional file 1: Appendix A. The respondents with scores in the highest and lowest ranges were sequentially selected for 45- to 60-min semistructured face-to-face or telephone interviews. The sample size was determined by data saturation; data were collected until no new information was gained [19]. We estimated that a sample of 15 to 20 interviewees from each group (the highest and lowest scorers) would provide sufficient data; actual saturation was reached after fewer than 10 interviews in each group. The interviews were conducted using the interview protocol in Additional file 1: Appendix B. The interviews were audiotaped and professionally transcribed.

### Data analysis

The survey data were analyzed using SPSS version 21.0 (IBM, Armonk, NY). Descriptive statistics were used to determine the distribution of the sociodemographic variables. Thematic analysis, as described by Braun and Clarke [20], was used to probe the interview data. All data coding was performed by one of the authors (A.E.) and reviewed by the second author (D.Q.) and the research advisors. Any coding disagreements were discussed and the analysis refined until consensus was reached. Qualitative data analysis was performed using the software MAXQDA version 11.1.0. A mixed-methods analysis was conducted by grouping the interviewees into sets based on their survey scores: a high-transfer group (scores 9–13) and a low-transfer group (scores 0–8). The score cutoffs were decided by consensus among the researchers. The characteristics of these groups were then analyzed in relation to the themes and subthemes that emerged in the interview data in order to understand how the high-transfer and low-transfer groups differed.

The Institutional Review Board of the institution where the research was conducted approved the study (Protocol PA14-0588). All survey participants read and acknowledged an electronic consent document. All interview participants provided consent for audiorecording before the interviews took place. The original data and interview materials will not be shared in order to maintain participants’ confidentiality.

## Results

### Survey results

**Fig. 1:**
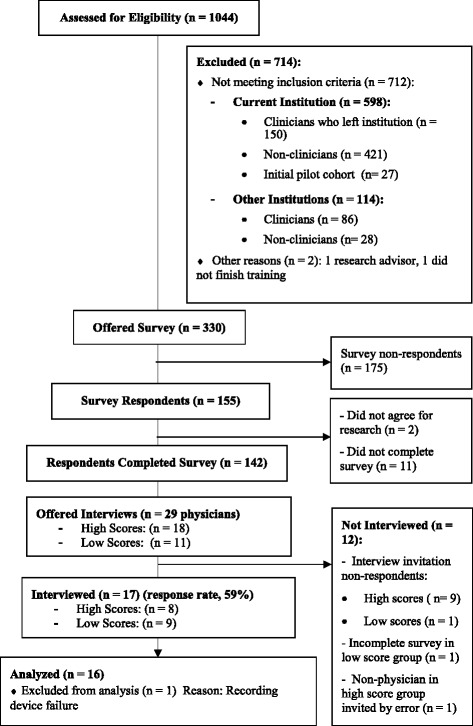
Study flow diagram

We identified 1044 trainees who participated in the QI training between 2005 and 2014. The recruitment email was sent to the 330 individuals who were still affiliated with the institution at the time of the survey. The survey was completed by 142 (43%) of the trainees who received it. Figure 1 shows the study flowchart. The majority of the respondents (*n* = 95, 67%) were between 40 and 59 years of age. Sixty-six percent were women. Most (88%) respondents had worked at the institution for more than 5 years. Physicians represented 49% of the respondents; nurses, 40%; physician assistants, 6%; and pharmacists, 5%. One-third (*n* = 47, 33%) of respondents were from cohorts that underwent the QI training between 2005 and 2009, and 95 (67%) respondents were from cohorts that underwent the QI training between 2010 and 2014. The Cronbach alpha for the survey was 0.89, confirming that the survey instrument was a sufficiently reliable measure of training transfer success.

### Interview results

Of the 142 survey respondents, 86 (61%) consented in advance to participate in a follow-up interview if selected. Because 51 (36%) survey respondents had the highest possible survey score, we decided to narrow our sample and interview only physicians in the qualitative phase of our study. This decision was made by consensus among the research group mainly for practical reasons, as we did not have the resources to interview all high-scoring participants. We also agreed that it was most important to learn about physicians’ experience with the QI training program because they usually assume leadership of QI projects in the institution. We invited 29 physicians whose survey responses placed them in either the high-transfer or low-transfer groups for interviews (18 high scorers and 11 low scorers); of these, 17 (8 high scorers and 9 low scorers) agreed to be interviewed. One interviewee withheld consent for the use of direct quotations from the audio recording. Sixteen of the 17 interviews were transcribed and analyzed; 1 interview was excluded from the analysis because the recording device failed.

The sociodemographic characteristics of the 70 physicians who responded to the survey and 16 physicians whose interview transcripts were analyzed are shown in Table 1.

**Table 1:** Sociodemographic characteristics of the 70 physicians who completed the survey and the 16 physicians interviewed and whose transcripts were analyzed

Sociodemographic characteristic	Physicians surveyed (*n* = 70)^a^ No. (%)	Physicians interviewed (*n* = 16)^b^ No. (%)
Gender		
Male	40 (57)	8 (50)
Female	30 (43)	8 (50)
Age in years		
Under 50	40 (57)	7 (44)
50 or more	30 (43)	9 (56)
Training cohort number		
Cohorts 1–10 (2005–2009)	28 (40)	6 (38)
Cohorts 11–21 (2010–2014)	42 (60)	10 (62)
Work experience at current institution in years		
Less than 10	29 (41)	7 (44)
10 or more	41 (59)	9 (56)
Academic role		
Professor	26 (37)	5 (31)
Associate Professor	24 (34)	5 (31)
Assistant Professor	17 (24)	4 (25)
Fellow	3 (5)	2 (13)

^a^Non-physician survey respondents excluded

^b^One physician transcript excluded due to recording device failure

**Fig. 2:**
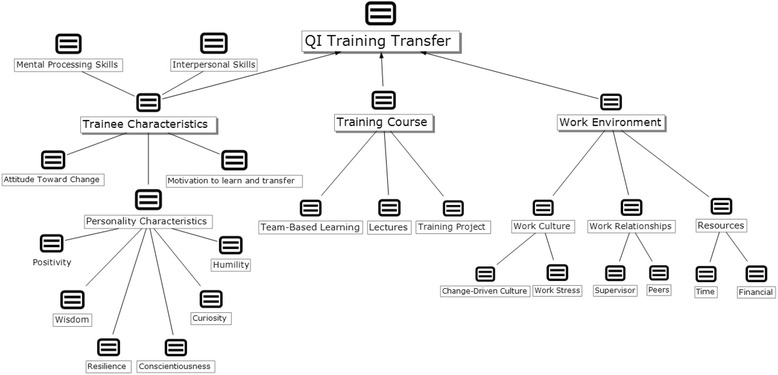
Factors and personality characteristics predicting quality improvement training transfer

As shown in Fig. 2, the qualitative and mixed data analyses showed 3 categories of factors found to predict QI training transfer: *trainee characteristics*, *training course*, and *work environment*. These themes were consistent with categories that have previously been identified in the literature. Within each of these 3 categories, several subthemes emerged from the thematic analysis. Five trainee characteristics were identified: *attitude toward change*, *motivation*, *mental processing skills*, *interpersonal skills*, and 6 *personality characteristics* (*curiosity*, *humility*, *conscientiousness*, *resilience*, *wisdom*, and *positivity*). The theme *training course* was divided into 3 subthemes: *training project*, *team-based learning*, and *lectures*. The subthemes of *work culture*, *work relationships*, and *resources* composed the category of *work environment*. The definitions of some selected themes and subthemes are shown in Additional file 1: Appendix C which shows that these themes and subthemes are the result of merging and grouping of different related codes. An example for that are the 3 personality characteristics *resilience*, *wisdom*, and *positivity* where *resilience* encompassed codes related to persistence, courage, and self-confidence; *wisdom* had attributes related to patience and maturity; and *positivity* consisted of codes related to acceptance and flexibility.

#### Trainee characteristics

While we identified some trainee characteristics that were consistent with those described in the extant training transfer literature, such as *positivity, motivation, and mental processing skills*, we also identified several new trainee characteristics that predicted successful QI training transfer: *attitude toward change*; *interpersonal skills*; and 5 personality characteristics: *curiosity*, *humility*, *conscientiousness*, *resilience*, and *wisdom* (Fig. 2). Sample quotations from interview transcripts that reflect these themes are given in Table 2.

**Table 2:** Trainees’ Quotes Representing Themes and Subthemes

Trainee Characteristics:
• Attitude toward change: - Positive attitude toward change: *“I think the people who are believers in change are the ones that tend to get the most out of the course and are most likely to apply their tools.”* —P227 - Conditional/situational attitude toward change: *“Although I would love to see this change happen, I’m one of the people who won’t do it because the way I like to do things is not—it will end up taking me as much time as to invest in letting someone else do it.”* —P303
• Motivation to learn and transfer QI skills: *“So I think it’s an internal motivation that quality is important, that safety is important.”* —P106 *“So my own set of motivations was how I could make the clinic experience where I am and the patient experience where I’m working better. And I knew that we would figure out a way to do that through the context of the course one way or the other.”* —P129
• Interpersonal skills: *“And you need somebody who can communicate and can sort of rally the troops behind your idea.”* —P190
• Mental processing skills: *“It was nice to kind of learn the tools to organize and analyze a problem and brainstorm solutions and how to label those solutions in different aspects of tackling a problem.”* —P152
• Personality characteristics: - Curiosity: *“If you’re not willing to be open minded and curious then you can’t really deliver care because things never happen the way that you think they’re supposed to.”* —P129 - Humility: *“I’m not a very creative person. What I think has been really wonderful on the teams that I’ve worked on is that everybody has different skill sets that they bring to the team.” —*P227 - Conscientiousness: *“When you look at the projects themselves, like when they pick the projects, they need to make sure that it’s really going to make a difference to the institution or to the patients or whatever they are looking at.”* —P028 - Resilience: *“I was always kind of taught in life, and somebody told me early on, that hurdles are formed for people that can’t get over hurdles. And I always kind of looked at things like that and if I run up against a situation and it doesn’t work for me, I can still be persistent.”* —P072 - Wisdom: *“I’m always of the kind of mind set that I need to try to make the right decision. And if somebody above me makes a wrong decision, that’s not my—that’s not my fault.”* —P072 - Positivity: *“I’ve had more projects fail because I didn’t have the right people in the committee, and all my failures were just as important as all the successful projects.”* —P248
Training Course:
• Project: *“I think a formal course is very important because you understand about process maps and those kinds of things. But I think that if you don’t connect that with a real-day project, it’s not helpful. It’s too theoretical.”* —P029
• Team-based learning: *“I think the team-based learning was very good… So that’s—that really helped me. And right now, like when I think about it, like process improvement, it’s like everybody working together, because everybody has their own qualities.”* —P028
• Lectures: *“The invited speakers really changed the way that I actually thought about how we deliver healthcare and sort of what we might think about doing moving forward.”* —P129
Work Environment:
• Work culture: - Change-driven culture: *“Well, we’ve had this culture for the past thirty years or twenty-five years, and it’s the same people. They have the same mindset. And they just don’t want to change. So it’s very difficult.”* —P028 “*I think our department is set up in a way that allows for the opportunity to bring new ideas to help change.”* – P227 “*We’re always looking for a change because we are a new specialty. Not many of us are around. We are—all of the faculty are—open for a change. If you get a new idea, they jump on it.”* – P237 - Work stress: *“This place is a wonderful place to work, but a lot of pressure and burden…. If I’m under stress, I don’t feel like doing the project I was supposed to do.”* —P234
• Work relationships: - Relationship with supervisor: *“You could have all the motivation. However, if there is no alignment with what your supervisors, your managers have, you might not go anywhere with it—with this motivation.”* — P106 - Relationship with peers: *“I think our group is fairly receptive to quality improvement projects. You know—there was initially some resistance, but I think all of us kind of know each other well enough where we know that we’re not trying to increase our own workload or make our lives more difficult.”* —P152
• Resources: - Time: *“The main resource I would say is time. It’s difficult to find the time to do these—to even go to these meetings, to come up with ideas and to organize ideas, and do data collection if we need to.”* — P152 - Financial resources: *“I think one of the goals should be figuring out how to do quality without having dedicated resources because I think it’s unrealistic in today’s climate that you will have anything you want at your fingertips. Learning to be resourceful is a skill that is necessary to make projects succeed.”* —P227

The outcomes of the comparison between the characteristics of trainees in the high-training-transfer and low-training-transfer groups supported the validity of the themes and subthemes. For example, in the subtheme *motivation to learn and transfer QI skills*, none of the participants in the low-transfer group (scores 0–8) had interview segments coded under this subtheme, but all the participants in the high-transfer group (scores 9–13) had interview segments coded under this subtheme. Another example was the personality characteristic *humility*, which we defined as a trainee’s having the attributes of humbleness and willingness to learn from others. While none of the participants in the low-transfer group had interview segments coded under this subtheme, all the participants in the high-transfer group had either exhibited this personality characteristic or talked positively about it in their interviews.

#### Training course design

We identified 3 themes related to the design of the training course that affected QI training transfer, as shown in Fig. 2: the *required project*, the use of *team-based learning*, and the *lectures* given during the training. Both the project and the team-based learning were seen as highly important by the interviewees, as illustrated in the following quotes:

To have a project that’s really meaningful helps because then everybody gets to work on it, and then they see the impact and they appreciate seeing something that’s changed and helped *–* P145

I think attending that course really opened my eyes about team dynamics and how—what you can learn from that. *–* P237

Other representative quotations from interview transcripts related to these themes appear in Table 2.

#### Work environment

We identified in our interview data 3 themes related to the work environment: *work culture*, under which we included the subthemes *change-driven culture* and *work stress*; *work relationships*, which we broke down into the subthemes *supervisors* and *peers*; and *resources*, which included the subthemes *time* and *financial* (Fig. 2). Some representative quotations related to these themes and subthemes are provided in Table 2.

## Discussion

Using a mixed quantitative (survey) and qualitative (interview) approach, we identified factors predicting training transfer in health professionals who participated in a QI training program at a large hospital in the United States between 2005 and 2014. While some of those training transfer predictors where identified in other studies some such as *attitude toward change*, *motivation*, *conscientiousness*, *mental processing skills*, *interpersonal skills*, *training course elements,* w*ork culture*, *work relationships*, and *resources* [8, 9, 13, 14, 16, 21–30] we identified different training transfer predictors such as *curiosity*, *humility*, *resilience*.

### Implications of the study

Our results will help organizational leaders and quality improvement officers to select the health professionals who are most likely to benefit from QI training interventions and to ensure that their work environments are conducive to QI training transfer. Because health professionals and health care organizations must invest significant amounts of time, effort, and funding in QI training, ensuring the optimal selection of trainees will maximize the positive effects of and return on investment from the training, especially considering the budget constraints many health care institutions currently face. We recommend selecting trainees for QI training programs based on the trainee attributes and personality characteristics identified in this study. One strategy for selecting trainees would be to administer personality inventories to potential participants in QI training. Another option would be to offer pre-training sessions to trainees who score low on measures of the modifiable characteristics, such as *motivation* and *positive attitude toward change*. Such sessions could help those trainees acquire the characteristics that predict training transfer success before they begin the QI training.

In future research, we plan to elaborate on 3 of the personality characteristics that have not often been identified in the training transfer literature as a whole: *humility*, *curiosity*, and *resilience*. We speculate that these characteristics may be particularly prominent in trainees who successfully learn and transfer QI skills. Humility helps successful trainees to reflect on their strengths and deficiencies and to be willing to make changes. Curiosity allows trainees to question the status quo and to seek explanations and solutions for existing process problems. Resilience helps trainees to confront resistance to change and overcome challenges impeding improvement. Therefore, we recommend selecting trainees whose personality analysis shows that they have high levels of humility, curiosity, and resilience.

Our study also identified the theme of *work culture* and its subtheme *work stress* as important factors predicting QI training transfer. In order to ensure organizational readiness for QI training transfer and to maximize the training’s benefits, leaders should establish a change-driven culture [25] and implement procedures to reduce work stress.

In addition to the personal and organizational factors predicting QI training transfer, our study identified factors related to the design of QI training courses, such as the *training project* and *team-based learning*. In our opinion, these findings have instructional implications that are beyond the scope of this article; they will be the subject of a future publication.

### Limitations of the study

Our study had several limitations. First, our screening survey was not psychometrically validated. We note, however, that despite the lack of formal validation, the survey was able to separate the respondents into 2 groups based on the degree of QI training transfer they exhibited. Second, the source of information about training transfer was exclusively the training participants themselves. This may have introduced subjectivity and biases into the data. Other sources, such as the trainees’ peers and supervisors and the training instructors, could also have provided valuable data. However, we were able to minimize, if not eliminate, this bias by designing the survey to measure the application of skills and production of deliverables instead of the trainees’ satisfaction with the course or their acquisition of specific knowledge. Third, our study had a small sample size and was restricted to physicians in a single institution, which may limit the generalizability of our results. Confirming the results of this study through studies of other categories of health professionals and types of institutions will be an important future step in research. Fourth, the data were gathered from individual and not group interviews. Valuable data about team characteristics and dynamics could have been gathered by interviewing teams (for example, through focus groups). Nonetheless, we believe that we collected enough data about team dynamics as well as participants’ perceptions of their teams from the individual interviews. Finally, by the time of the semi-structured interviews, up to 9 years had elapsed since some of the participants completed the QI training. This time lag may have decreased the accuracy and completeness of the collected data, as other QI training experiences may have happened in the interim. This was encountered with only 1 senior participant in the high-score group who participated in the sixth cohort of the training and who had taken QI training courses at other institutions in the past. However, we think that the time lag had only a limited effect on our study results because we identified several personality traits, which are unlikely to change over time, among the factors predicting training transfer. In spite of these limitations, our novel study adds new and unique training transfer predictors to the extant literature. In addition, our results will help leaders of health care organizations to develop methods for selecting the trainees who are most likely to transfer QI training and ensure that work environments are conducive to QI training transfer.

## Conclusions

To our knowledge, this is the first study aimed at identifying the factors and personal attributes predicting QI training transfer among health professionals. Our results add to the existing research in the area of QI training transfer. While some factors in QI training transfer are likely universal to any training, we speculate that others are more relevant to QI, such as attitude toward change, personality characteristics (curiosity, humility, and resilience), and change-driven work culture. Our results also show that in order to ensure organizational readiness for QI training transfer, leaders should establish a change-driven culture and implement policies that reduce work stress. We anticipate that the results of our study will help organizational leaders select health professional and physicians who are most likely to transfer QI training which will ultimately contribute in enhancement of health care QI initiatives.

Future research should explore the role of teams in training transfer. Group data are needed in addition to individual data. Future studies should also aim to identify the factors and attributes that relate to QI training transfer among health care professionals other than physicians and compare them to the results of this study.

## Additional file

Additional file 1: Appendix A. Survey Questionnaire. Appendix B. Interview Protocol. Appendix C. Definitions of Selected Themes and Subthemes. (DOCX 30 kb)

## Abbreviations

QI: Quality improvement
