# Supplementary material for: Factors predicting training transfer in health professionals participating in quality improvement educational interventions
Source: BMC Med Educ. 2017 Jan 31;17:26. doi: 10.1186/s12909-017-0866-7 (PMC5282779; doi:10.1186/s12909-017-0866-7)
Supplement: Additional file 1: — Appendix A. Survey Questionnaire. Appendix B. Interview Protocol. Appendix C. Definitions of Selected Themes and Subthemes. (DOCX 30 kb) [file 12909_2017_866_MOESM1_ESM.docx]

**Additional file 1: Appendix A. Survey Questionnaire**

1. I have used the QI training tools (Fishbone diagram, Flowcharts, Pareto charts, Run/Control charts) and/or methods (Team effectiveness, Customer focus, PDSA cycles, Completing a project) I learned in the QI training to conduct improvement in my practice.

a) Yes, with clearly positive results. (4 points)

b) Yes, but I haven’t experienced any discernible results yet. (3 points)

c) No, not yet, but I expect to. (2 points)

d) No, and I do not expect to. (1 point)

e) Not applicable. (0 points)

2. I have used the QI training tools (Fishbone diagram, Flowcharts, Pareto charts, Run/Control charts) and/or methods (Team effectiveness, Customer focus, PDSA cycles, Completing a project) I learned in the QI training to conduct improvement in my department.

a) Yes, with clearly positive results. (4 points)

b) Yes, but I haven’t experienced any discernible results yet. (3 points)

c) No, not yet, but I expect to. (2 points)

d) No, and I do not expect to. (1 point)

e) Not applicable. (0 points)

3. Which of the following best represents the extent to which you have implemented other QI projects based on the knowledge and skills you acquired from the QI training (different than the project required for the QI training)?

1. I have completed a QI project that has since been put into action with a positive result. (5 points)
2. I have completed a QI project that has since been put into action but I haven’t

experienced any discernible results yet. (4 points)

1. I have completed a QI project, but it has not been put into action yet. (3 points)
2. I have started a QI project, but it isn’t complete yet. (2 points)
3. I intend to develop a QI project, but haven’t started yet. (1 points)
4. I have no intentions of developing a QI project. (0 points)

**Additional file 1: Appendix B. Interview Protocol**

**Topic Domain 1: Training Issues**

**Start-off Question:** Please tell me about your experience with the QI training program and how you got involved with this program.

**Follow-up Questions:**

1. People say different things about the usefulness of health care professionals training in general and QI training in particular? What do you think about that?
2. What were your expectations from the QI course before you attend it? The QI course uses different instructional methods such as didactic lectures, project, team based learning… etc. Can you think of any that were helpful to you?
3. If someone you know is taking the QI course, what recommendations you would make to that person?
4. If you are the one planning and running a QI course, what would you do? Out of the things you mentioned what is currently done?
5. a- If you are selecting trainees for the QI course, what individual characteristics you would base your selection upon to maximize the training application?
6. You mentioned ….. (e.g. curiosity, discipline, bravery…etc.). Would you please tell me how you define these and please give me an example from your life or someone else’s life that explains these attributes?
7. How much are you currently involved with QI activities? If not currently involved, when did you stop being involved, what were the reasons for that, and how did you feel about that?

**Topic Domain 2: Work Environment Issues**

**Start-off Question:** How would you describe your practice and department work environment?

**Follow-up Questions:**

1. In your opinion, are resources an important part of applying what is learned in the QI course? Can you please give me an example? How does that apply to your QI training?
2. What happens when someone does (or tries?) things to improve her performance in your department/practice? What is your personal experience when you try to do things?
3. How ready are people in your group to change the way they do things?
4. How do you describe your workload in regards to allowing you to try the new things you have learned?
5. Can you think about someone who took the QI course and made a change in her practice or department? In your opinion, what in that person’s environment influenced implementing what was learned in the QI Program? How does that apply to you?

The interviewees were thanked and asked if there was anything else they would like to tell us or thought we needed to know.

**Additional file 1: Appendix C. Definitions of Selected Themes and Subthemes**

**Trainee’s attitude toward change:** The degree of the trainee’s favorability toward change and the degree of the trainee’s belief in change.

**Positive attitude toward change:** High degree of favorability toward change.

**Conditional/Situational attitude toward change:**  Changing degree of favorability to change depending on conditions and situations.

**Early adopter:** High degree of intention to adopt change.

**Late adopter:** Low degree of intention to adopt change.

**Not early or late adopter:** An intermediate degree of the trainee’s intention to adopt change.

**Personality characteristics:** The relatively stable attributes of individuals that influence their cognition and behavior.

**Curiosity:** Attributes of open-mindedness and ability to reflect on self and others.

**Humility:** Attributes of humbleness and willingness to learn from others.

**Conscientiousness:** Attributes of being organized, responsible, efficient, committed, and focused

**Resilience:** Attributes related to persistence, courage, and self-confidence.

**Wisdom:** Attributes such as patience and maturity.

**Positivity:** Attributes related to acceptance and flexibility

**Interpersonal skills.** Effective communication with others and team building skills.

**Mental processing skills.** Evidence-based thinking, problem solving, critical thinking, maintaining focus, and creativity.

**Work culture**: Values and behaviors that contribute to the unique social and the psychological environment of the trainee’s department or institution.

**Change-driven culture:** A culture that is conducive to change and quality improvement.

**Work stress:** The harmful emotional response that occurs when the requirements of the job do not match the needs of the trainees; associated by some participants to the organizational and/or departmental work culture.

**Work relationships:** The professional relationship between the trainee and others at work, such the trainee’s supervisor and peers.

**Resources:** Factors related to the availability of resources that enable training transfer, such as time, human resources, and financial resources.
